# An Introductory Lecture Delivered at the Massachusetts Medical College

**Published:** 1848

**Authors:** 


### Part 3.—Bibliographical Notices

ARTICLE I.

*An Introductory Lecture delivered at the Massachusetts Medical College, Nov.* 3, 1847. By Oliver Wendell Holmes, M.D., Parkman Professor of Anatomy and Physiology. Boston: W. D. Ticknor, & Co. pp 38. Copy right secured.

As a literary production, this address is entitled to all the praise that was bestowed upon it in Boston, about the time of its delivery and publication; but it is evident that the author's ideas of the West and South are very limited, as his opinions of the medical profession and the Medical Schools in these regions, are very erroneous. It will be amusing to our readers, (for we are sure they know how to make allowances for it, so as not to take umbrage,) to hear the sweeping denunciations against them, and the schools which it is their interest and pride to foster and build up, made in this maiden effort of Dr. Holmes, as a professor; and the complacency with which he speaks of Boston and the school with which he is connected.

We hope ere he holds forth on the subject again, he will take occasion to visit the "great West" and South, and pass from Chicago to New Orleans, visiting the points enumerated in the article quoted below, as the sites of Medical Schools, making the acquaintance of members of the profession on his route; and our prediction is, that he will not be able to give a history of what he has seen, and an account of the greatness to which the country, and of medical men and medical schools, are destined, without becoming as eloquent in our praise, as he has been severe in our condemnation.

There is no doubt that as the Mississippi valley becomes the seat of empire for the Union, and the principal place of commerce and manufactures, as it is of agriculture already, that the medical profession will also take the high stand, to which it is now marching forward, of pre-eminence, occupied by the country in other respects. A glance at the progress of the last few years, in building up institutions of learning, and a view of the almost unbounded capacity of our country, point with unerring certainty, to the time when our halls of learning will be crowded, not only with students from all the States of the Union, but from all the civilized countries in the world.

As our talented friend Prof. Flint has shown up in their true light these denunciations and high pretensions, in a spirit of candor, and in a manner to meet our entire approbation, we quote from the Buffalo Medical Journal, his remarks in reference to the address.

"Dr. Holmes is favorably known as author of some valuable communications on medical subjects. He enjoys greater celebrity, however, in the walks of polite literature. He furnishes an illustration of the compatibility of pursuits so antagonistical, apparently, as physic and poetry, having cultivated the latter with a degree of success which might well satisfy a reasonable ambition. But the annals of medicine are not without examples in which the characters of poet and physician have been united, and preserved, without deterioration of either character; and Apollo, if our mythological knowledge serves us, was the Divinity of the lyre, as well as of medicine. The lecture, as a literary effort, takes precedence of all which we have received; but, as regards matter and tone, we must say, it seems to our prosaic apprehension to be open to criticism. Having said this much we are bound to adduce some of the portions of the lecture to which our remarks have reference.

"Having alluded in terms of approbation to the organization of the National Medical Association, the lecturer speaks of its objects, &c., in this wise :

"'If the feeling in which this originated were truly stated, it would be found that it was a result of the institution of *inferior medical schools*, situated in the *wrong places*, and managed by the *wrong men*, and the consequent cheapening and *vulgarizing* of education, until the degree of doctor of medicine has, in *some parts* of our country, ceased to be an evidence of a decent amount of knowledge on the part of those who possess it. The honorable and thorough bred practitioner found himself shouldered by ignorant novices claiming to be his equals, who had never devoted half the allotted period of pupilage to study, who had never touched a scalpel, who had never *seen* a hospital, and in the face of an easy public, apt to take men at their own valuation, and having no proper means of discriminating between them.* Such, I believe, was the original mainspring of the movement, but other and more useful designs have been superadded to the principal one of raising the degenerated standard of education. Among them are the thorough union and organization of the profession, the establishment of an elevated code of ethics, and the pursuit of various inquiries in concert by physicians of different sections of the country.

"'There are two great objects, then, aimed at by the National Medical Association: one may properly be callled *Reform;* it implies the previous existence of abuses, and casts a reproach upon all to whom its measures apply, for doing some wrong, or neglecting some duty. The other is *improvement*, and this it may be presumed every body and association in the country, like every individual person, is capable of, and will find ample room for, if it is to be urged upon them. Now, I believe I may say with confidence, that neither this community nor this institution are obnoxious to any charge that calls for the agency of *reform* as that word is commonly intended. We *reform* the victims of habitual intoxication, but if we induce a strictly temperate person to become more severely abstinent, we may call it improvement if we will, but not reformation. Our medical community was already thoroughly organized; an ethical code, in most respects identical with that adopted by the National Convention, had been in action here for a long series of years; a general state of harmony and an enlightened public sentiment were already prevalent. In this University there was a full complement of instructors; the first of all requisites, clinical teaching, was thoroughly attended to, and the full period of study, as I once learned to my infinite inconvenience, always rigorously insisted upon.

"'Let us distinguish, then, between reform and improvement. We can all improve, let us hope that we do not all stand in need of reform. The word becomes offensive and impertinent when used too freely. It has the effect of the crack of a whip in a pasture full of quiet and contented animals. The peaceful creature who was solely intent upon

* It is a little remarkable that on the morning after these words were written, I received a letter from one of the great States of the distant West, containing the following sentences, "We have several miserable apologies for medical schools in this State, which by cheapening the rates of instruction induce many young men of limited means to attend their lectures. The consequence is that we have many among us who flourish an M.D., and yet are ignorant of the elementary principles of Medical Science."

his thistle, looks up and gives loud utterence to his impressions, and the unshod colts throw up their foolish heels into the air as if there were to be an end of all slow coaches from the date of their superfluous gambols.'

"If a feeling resulting from 'the institution of *inferior medical schools* situated in wrong places,' &c., originated the 'great national project' of the Convention, it is truly surprising that the proceedings of the Convention do not contain even an allusion to the subject. Strange that no action was had upon it, and that no member of the Convention even proposed it as a matter for deliberation! We concur, however, in the propriety of characterizing the objects of the Convention as objects of *improvement* rather than *reform*, but, with all our predilections for our *alma mater*, we are at a loss to perceive the propriety of assuming that the distinction applies *par excellence* to the Boston School. We have contended, on a previous occasion, that as applicable to medical institutions, and the medical profession of the country generally, the terms improvement, and progress, are more appropriate than reform. It were easy to show from the history of both a continued career of advancement—neither have receded in excellence and usefulness, but the course of each has been steadily onward—how, then, can they be said to need reform. They are both capable of being bettered, and to combine and concentrate efforts for this end, we presume, was the prime purpose for which the convention was projected and carried into effect. In pursuance of this end the Convention adopted resolutions, recommending certain improvements in the system of instruction at medical schools. Now in these recommended improvements had the Boston School anticipated the action of the Convention, so as to render this action inapplicable to it?

"Dr. H. says: 'In this University there was a full complement of instructors.' Would it not have been more strictly correct to say there *is* a full complement, for, if we mistake not, the number recommended by the Convention, viz., seven, did not exist at the time the first Convention was held? It was then but six, anatomy and operative surgery being taught by the same professor. It has since been increased to eight. It was recommended by the Convention that dissections be rendered obligatory upon pupils before graduation, yet, we find by the reply to the interrogatory whether at that school the student is required to dissect, that it is voluntary with the student. Some other Institutions have already adopted this improvement. Again, the Convention recommended that Medical schools take measures to insure constant attendance of pupils at lectures. Is this done at the Boston School? And, again, as respects the still more important improvement of extending the lecture term to six months, we have yet to be informed that our venerated *alma mater* has followed the example of other schools in acceding to this improvement. We shall surely not be accused of a desire to depreciate that institution of all others, but we must say that we are at a loss to perceive that it differs from a majority of the *well located* medical institutions of our country so far as to be excepted from any application of the recommendations of the Convention.

"In the quotation which follows, the writer reiterates the stereotyped complaint of the multiplication of medical schools, and the pernicious effect of competition. The reader will, of course, form his own opinion of the taste evinced in the allusions to other teachers, which is indulged in an introductory lecture by a newly appointed professor. As respects the effects of competition in Medical schools, we can perceive no reason why it should not have its advantages here, as in every other sphere of effort. We know it is customary to attribute most of the evils under which the profession labors to this source, but we believe no reason can be assigned for the opinion except that it has become habitual. We cannot devote space, in this connection, to the discussion of the topic, but we would simply ask two or three questions, to direct the attention of our readers to facts which settle the matter practically and fully: Is the University of Pennsylvania less deservedly distinguished in its educational advantages than it probably would have been, had the Jefferson school never been organized? Has the Jefferson school suffered by competition with the several other institutions which have been more recently established in Philadelphia? What was the condition of the College of Physicians and Surgeons of the city of New-York before the University school went into operation, and what is its comparative reputation now? If our Boston friends will reflect upon these questions, we honestly believe they would not be alarmed at the prospect of a rival medical school in their own city. Excellent as the school now in existence there is, it would become, in our opinion, better, and more flourishing under the stimulus of competition. We state this as a prediliction, for the time is probably not far distant when the experiment will be practically tried. But to the quotation which has suggested these desultory remarks:

"'The extreme and rapid multiplication of medical schools has certainly had its use in educating the teachers, if not the students, and perhaps in giving a little training to some of those who might have gone into practice without any. I

need not say what bad consequences it has had; I have hinted at them already, and the country is full of loud complaints on the subject. So long as the moderate compensation of the teacher is an inducement to unemployed young men; so long as the poor title, bestowed upon every itinerant who juggles a living out of the pseudo-sciences, can tickle the ears of the half-taught practioners, will legislatures be teased to grant charters for new schools, not only uncalled for by any public want, but tending directly to lower the standard of education. Competition is a good thing in its place, but it has blown up hundreds of poor wretches who trusted themselves in *Western* steamboats; and if suffered to run riot through our medical institutions they will be true to the parallel of hot fires, thin boilers, close valves and their inevitable consequences. There is a natural fitness of circumstances that might be regarded with some advantage. Men do not establish factories on hill-tops, where the water that turns the wheels must all be pumped up by hand. They do not organize infant schools in diving bells, where the light is to be let in through a bull's eye, and the air to be sent down in demijohns. Rivalry in medical institutions must always exist, but this business of underbidding and under-feeding, this farming out of medical students, like town paupers, to the lowest contractor, must eventually be arrested. If the law cannot do it by the necessary discrimination, organized public opinion can and will do it. And the time must come when those institutions which cannot by any possibility afford practical instruction in the most important branches of the profession will cease to be recognized as capable of giving a full title to public confidence. It is but the addition of three or four letters to those which designate the medical gratitude, and the *Doctor Medicinæ Pennsylvaniensis* or *Harvardiensis* is as well known as the Parisian graduate by the title which he never fails to claim, and the equality which now confounds the most important differences is at once overthrown and abolished!'

"Our readers have not failed to observe from the tenor of a note annexed to the extract preceding the last, that Dr. Holmes entertained a very low opinion of Western Schools. His ideas of Western and Southern teachers may be gathered from the extract which will follow. His remarks upon multiplied schools, and 'inferior medical schools located in wrong places,' 'unemployed young men becoming teachers for the moderate compensation,' &c., seems to have reference especially to the West and South. We cannot let this pass without a remark or two, although our Western and Southern brethren are abundantly able to defend themselves, and perhaps will not thank us for volunteering any comments on this attack, for so we must term it. Our remarks will be rarther apologetic for the writer than otherwise. We have some little practical acquaintance with 'Boston notions.' One of these notions is, that Boston is the *ne plus ultra* of all places, another is, that it is the *sine qua non*. We should be sorry to seem obnoxious to the accusation of being wanting in respect for the metropolis of our native state. The accusation would be groundless. We delight to honor it as the seat of learning, science and refinement; but it does not absorb all the talent, acquirement, and intellectual resources of the country.

"It is really amusing to a visitor at New England to observe how little all that that pertains to the 'great States of the distant West' (to quote the writer's expression) is appreciated. The Bostonian thinks it passing strange that in Europe his white skin occasions surprise. The ideas which prevail in New England respecting the West, appear not less strange to the citizen of the latter. The author of the address before us, in the remarks to which we have directed the reader's attention, only expresses a common sectional sentiment. We do him the justice to believe that he supposes his remarks to be such palpable truisms, that no offence can possibly be taken, for, we presume he would not knowingly violate the rules of courtesy and good-breeding, to say nothing of medical ethics. That he honestly thinks the medical institutions of the West are 'miserable apologies for medical schools' we have no doubt, and furthermore, it is to his mind so obvious that it must be so, as to require no reserve in uttering the opinion. He could not probably be induced to speak disparagingly of the schools which are, comparatively, in his neighborhood, viz., Woodstock, Castleton, Hanover, Bowdoin, all located in small villages. These are in New England, and cannot be charged with being 'inferior schools wrongly located,' the chairs filled with 'unemployed young men,' &c. New England needs them all. But in the 'great States of the distant West' and South, what are the schools referred to? They are as follows:—One at Chicago, Illinois, a town now larger than was Boston when the Boston Medical School was instituted; one at Laporte, in the great State of Indiana; two at St. Louis, a city larger than was Boston twenty years ago; one at Cincinnati, one at Columbus, and one at Cleveland, these from the great State of Ohio; two in Kentucky, one at Lexington the other at Louisville; one in Tennessee; one in Louisana, at New Orleans; one at Charleston, South Carolina. These are the Medical Institutions of the distant West and South. We beg the reader to cast his eye upon the map of the United States, and compare the territory over which these schools are scattered, with the area of New England with its seven Medical Colleges, (five of which are in villages) and more in contemplation !

" We will not enter upon the delicate undertaking of instituting comparisons of individuals, but there can be no invidiousness in enumerating some of the teachers connected with the 'miserable apologies for schools' in the distant West and South. The names of Drake, Caldwell, Gross, Dudley, Bartlett, Brainard, Linton, Blake, Delamater, Mussey, Lawson, Carpenter, Ackley, Dickson, Moultrie, Harrison, and others, are certainly familiar, not only at the South and West, but with tbe Medical Profession of the whole country.

" We must waive father remark, and after presenting the extract already referred to, proceed to notice some introductory lectures emanating from Western Schools, of which we have several on file.

"'There are many peculiarities in the medical character of this section of the country. Our position in New England, a little out of the broad current, our distinct origin, our hereditary habits, manifest their influence in the shades of professional as well as political character. We may expect to find the New Englander as cool, as shrewd, as practical in medicine as in business. But his peculiarities are best displayed in the medical teacher and the medical public. The first is singularly calm, simple and didactic, as compared with many of his distant brethren ; the second cautious, sedate, respectful to a degree which the fiery children of the South would call tame and submissive. When the annual flowering of 'Introductory Lectures' takes place, it may be seen that the colors are generally higher as the distance from the equator is less, and that the gayest display is from those that have had the advantage of the last rays of the setting sun ; the efflorescence of scientific enthusiasm on the banks of the Mississippi or Missouri. Whether it be the coldness of Northern winds or the sterility of Eastern soil, there is less leaving out in proportion to the yield, and that of a less glaring aspect in our sober nurseries of knowledge than in those of our Southern and Western friends. Our danger is in the direction of *sensible dulness*, and theirs in that of glittering wordiness.'"

We have had abundant opportunity to observe the character of our profession in the West ; and although we have to deplore the fact that there are many practising medicine without a good education, and without having attended lectures in any medical college; yet, it is not the case that *graduates* in medicine from western schools, are generally inferior in point of professional knowledge to those from the East. Not only so, but it is a fact that those educated in the West, at schools affording clinical instruction, go into the field of practice with decided advantage over those from the East, on account of their familiarity with the most common types of disease here, with which the eastern graduate must become practically acquainted, after he comes to the country. Of the truth of this, almost every physician from the East who has been in practice in the West, will abundantly testify.

From the Southern Medical and Surgical Journal, we have the following:

"We have, comparatively speaking, no *Parisian* graduates among us, and we deny that a Diploma from *Pennsylvania* or *Harvard* confers any distinction whatever upon its possessor. On the contrary, a graduate of the Medical College of Georgia requires no additional examination for license to practice in this State; but an applicant, especially from one of the New England Medical Colleges, (*Harvard* included) is subjected to a rigid scrutiny. And, why? Because of their imperfect organization, several of the schools having, until recently, but four or five professors."

It will be observed that the quotation in reference to which the above paragraph was written, does not, other than by implication, set up claims for the Harvard and Pennsylvania schools above all others. The manner and connection in which a simple truth is stated, are objectionable. The truth that a want of means to give clinical instruction renders an institution inferior to those that possess them, will scarcely be denied by any one; but that the schools of the South and West are less capable, generally, of affording clinical instruction than those of the East, is by no means so apparent. The facilities for giving clinical instruction, may be classed under two heads—1. The number of patients presented for observation. 2. The facility with which the student is afforded opportunities of examining them. In reference to the 1st, no one doubts that the advantages are greater at the East than the West. This depends upon the number of the pauper population, which is generally greatest in the largest cities. But the 2d depends upon the number of patients compared with the size of the medical class receiving instruction, and the liberties given them by hospital rules; and in this respect we apprehend the West and South will be found to afford equal advantages to the East, if not greater. Comparatively few students have the opportunity in the eastern institutions, where the classes are large, of making examinations for themselves, of cases under investigation; and without feeling the pulse, applying the stethoscope, making percussion, examining closely the tongue, the eyes, the countenance, feeling the skin, or examining the evacuations, they hear the clinical instructor prescribe. Can the student learn much more in this way, than by simply hearing the symptoms explained, and the treatment laid down in the college lecture room? Hundreds of our readers who have set at a distance of perhaps (very fortunately) only 20 or of 50 feet from the patient in a crowded amphitheatre, and *seen* the examinations and heard the prescriptions made, can say whether it gave them practical tact in the sick room or not, and whether a fewer number of cases closely and properly examined, would not have qualified them better for practice.

In most of the schools and hospitals of the West, the number of students in proportion to the patients and teachers not being so great, they are afforded more opportunities of walking the wards, and those having charge of hospitals, we believe, are more liberal in allowing privileges. In the Chicago hospital last winter, the class was divided into sections, one of which visited the wards daily, and each student was allowed to feel the pulse, auscultate, &c., &c., as the examinations were made.

